# Effects of high doses of selenium on the antioxidant status after liver resection

**DOI:** 10.1186/cc9794

**Published:** 2011-03-11

**Authors:** O Obukhova, S Kashiya, E Gorojanskaya, J Chekini, S Sviridova

**Affiliations:** 1 N.N.Blokhin Russian Cancer Research Center, Moscow, Russia

## Introduction

Selenium (Se) levels in serum for patients with colorectal liver metastasis are significantly lower than normal. The use of standard doses of Se has no effect on serum concentrations of Se or the antioxidant status (AS) indicators. It is assumed that the use of high doses of Se for patients undergoing extensive liver resection can improve their condition by enhancing antioxidant protection. The objective of this study was therefore to evaluate the effect of high doses of selenium on AS indicators, biochemical markers of hepatic failure and treatment results.

## Methods

Forty patients (M:F = 18:22, mean age 56) who were due to have a liver resection for metastatic colorectal carcinoma were recruited and were randomized into two groups. Patients of group 1 (G1, *n *= 20) received standard perioperative therapy. Patients of group 2 (G2, *n *= 20) additionally received sodium selenite according to the protocol: 2 mg on the first postoperative day, 1 mg in the next 4 days. The concentration of Se in serum, biochemical parameters (total bilirubin, AST, ALT), AS (toxic metabolites of nitrogen oxide (NOx), superoxide dismutase (SOD) and malondialdehyde (MDA)) and clinical data were assessed before surgery and on the fifth day after surgery. The significance of differences was assessed by Student's *t *test and the chi-square test.

## Results

There were no differences in the concentrations of biochemical markers of hepatic failure, duration of hospitalization, and 28-day survival after surgery. Before surgery Se levels were low (75.8 ± 8.7 vs. 72.8 ± 3.9). The NOx, MDA and SOD levels were elevated (respectively 35.1 ± 1.2 vs. 35.2 ± 1.8; 6.4 ± 0.4 vs. 6.6 ± 0.38; 106 ± 8.7 vs. 107 ± 8.8). After Se supplementation, Se levels were significantly higher in G2 compared with G1 (90.8 ± 7.42 vs. 75.7 ± 9.91, *P *< 0.05). On the fifth day the NOx, MDA and SOD levels decreased in G2 compared with G1 (respectively 29.5 ± 1.2 vs. 39.3 ± 2.2; 6.59 ± 0.9 vs. 9.8 ± 1.2; 84 ± 10.1 vs. 123 ± 7.7, *P *< 0.05). In G2, postoperative encephalopathy was significantly less (*P *= 0.013).

## Conclusions

Even in the early postoperative period, administration of high doses of sodium selenite in patients with colorectal liver metastasis who underwent extensive liver resection helps to improve AS. However, a small number of observations does not allow one to assess accurately the clinical effect of high doses of Se for these patients.

